# 656. Cryptosporidiosis in Immunocompromised Patients with Cancer Receiving Chemotherapy, CAR-T Cell Therapy and Hematopoietic Stem Cell Transplantation: A Retrospective Analysis

**DOI:** 10.1093/ofid/ofad500.719

**Published:** 2023-11-27

**Authors:** Inderjit Mann, Pablo C Okhuysen

**Affiliations:** Renaissance School of Medicine at Stony Brook University, Blue Point, New York; MD Anderson Cancer Center, Houston, Texas

## Abstract

**Background:**

Cryptosporidium is a parasitic protozoan that causes severe and potentially life-threatening diarrheal disease in immunocompromised patients. Infections can be asymptomatic, cause self-limited disease, or chronic diarrhea and in some cases result in biliary and pulmonary disease

**Methods:**

We performed a detailed retrospective analysis of patients that presented with diarrheal illness, and Cryptosporidium infection diagnosed by GI multiplex (Biofire) at UT MD Anderson Cancer Center between January 2017 and March 2023.

**Results:**

We identified 51 patients aged 24 to 89 years with cryptosporidiosis. Hematological malignancies (HM) were present in 44% of patients, while non-hematological malignancies (NHM) in 55%. Thirty-seven percent of patients had received hematopoietic stem cell transplantation (HSCT), 8% chimeric antigen receptor T-cell (CAR-T) therapy, 18% immunosuppressive therapy, and 65% received chemotherapy in the last 90 days. Two patients had human immunodeficiency virus (HIV). All patients presented with diarrhea, 39% with nausea, 43% with abdominal pain, and 51% had fever. The median duration of symptoms was 3 days. About 41% were severely neutropenic, and 61% had severe lymphopenia. Computed tomography (CT) abdomen was normal in 43% of patients, 16% had colitis, 12% had enteritis, and 4% had enterocolitis. All patients were treated with Nitazoxanide, with a median duration of therapy (DOT) of 14 days. The DOT was longer in HM than in NHM (14 ± 7.9 vs. 9.9 ± 5.6, p= 0.016) and in HSCT/CAR-T patients than without HSCT/CAR-T (13.8 ± 6.1 vs. 10.3 ± 7.3, p = 0.009). About 18% of patients had co-infections with other enteropathogens. Death from other causes occurred in 13 of 51 (23%) of patients. All cause mortality between HM and NHM (p=0.207), HSCT/CAR-T and non-HSCT/CAR-T (p=0.52), or chemotherapy vs. without chemotherapy (p=0.34) was similar. Most infections were encountered in the month of October.

**Figure:**
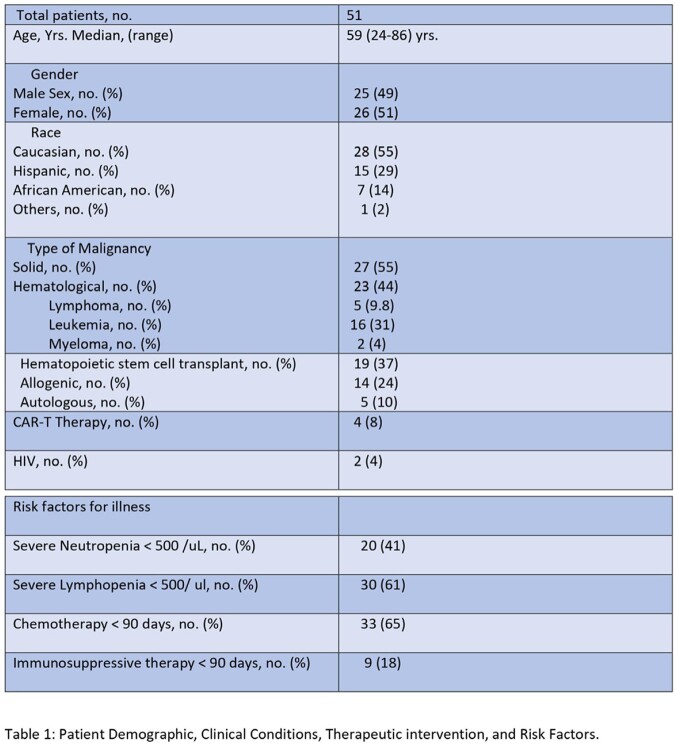

**Figure:**
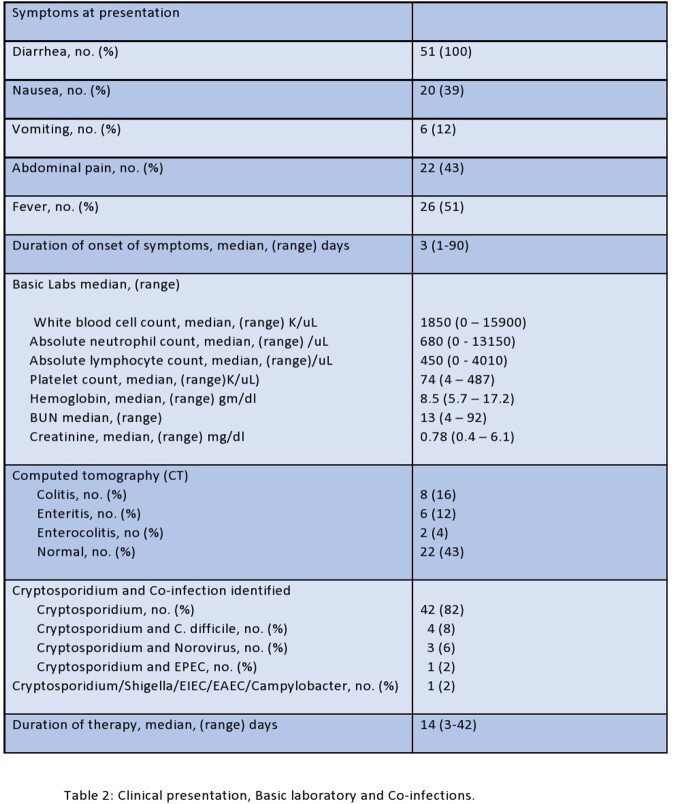

**Figure:**
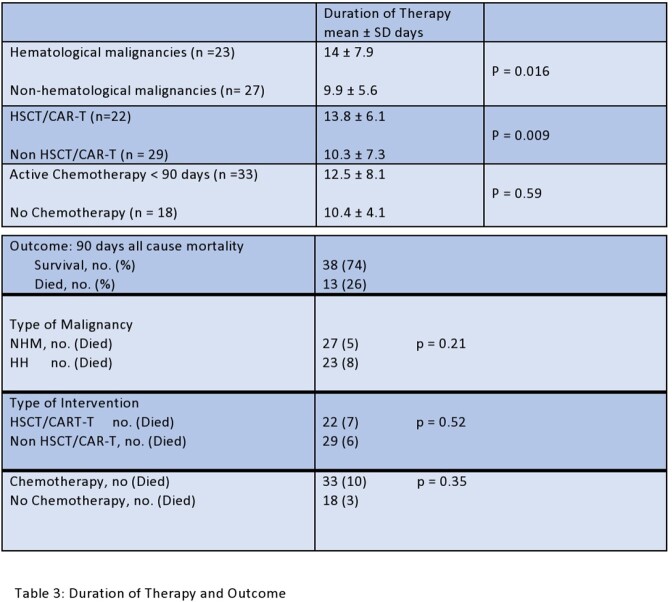

**Conclusion:**

We observed a limited response to nitazoxanide at our center, contributing factors and confounders included co-infections with other pathogens, active chemotherapy, or HSCT/CAR-T induced intestinal epithelium injury. Controlled, double blinded studies with new agents are needed to determine the optimal management of cryptosporidiosis in this population.

**Disclosures:**

**Pablo C. Okhuysen, M.D., FACP, FIDSA**, Astra Zeneca: Stocks/Bonds|Beam Therapeutics: Stocks/Bonds|Biontech: Stocks/Bonds|Deinove Pharmaceutucals: Grant/Research Support|Ferring: Advisor/Consultant|GSK PLC: Stocks/Bonds|Haleon: Stocks/Bonds|Johnson and Johnson: Stocks/Bonds|Melinta Pharmaceuticals: Grant/Research Support|Merk Sharp and Dohme: Grant/Research Support|Moderna: Stocks/Bonds|Napo Pharmaceuticals: Advisor/Consultant|Napo Pharmaceuticals: Grant/Research Support|Novavax: Stocks/Bonds|Pfizer: Stocks/Bonds|SNIPR Biome: Advisor/Consultant|Summit Pharmaceuticals: Grant/Research Support

